# From childhood trauma to psychosis: Investigating the attachment link

**DOI:** 10.1192/j.eurpsy.2024.1545

**Published:** 2024-08-27

**Authors:** S. Walha, I. Chaari, F. Guermazi, I. Dhouib, L. Aribi, F. Charfeddine, N. Mseddi, J. Aloulou

**Affiliations:** Psychiatry « B » department, Hedi Chaker University Hospital, Sfax, Tunisia

## Abstract

**Introduction:**

Childhood trauma encompasses instances of sexual, physical, and emotional abuse, along with neglect experienced during childhood and adolescence. Individuals with psychosis, particularly those with schizophrenia, exhibit a heightened prevalence of childhood trauma. One potential mediator in understanding this connection is insecure attachment.

**Objectives:**

This study aimed to better understand how childhood trauma relates to schizophrenia by examining two aspects of attachment: attachment anxiety and attachment avoidance.

**Methods:**

We conducted a descriptive and analytical cross-sectional study among stabilized female patients with schizophrenia or schizoaffective disorder, in the ‘B’ psychiatry department at Hedi Chaker University Hospital in Sfax, Tunisia, from May to June 2023. We administered the 26-item Revised Psychosis Attachment Measure (PAM_R) questionnaire, translated into Arabic, to assess attachment. Additionally, participants completed the 28-item Childhood Trauma Questionnaire (CTQ). We used both the Wilcoxon test for paired samples and the Spearman correlation test to assess the statistic differences and correlations.

**Results:**

We included 41 female patients, of which 65.9% had schizophrenia and 34.2% had schizoaffective disorder. The average age of the participants was 49.19 years. Among the attachment styles, avoidant attachment was the most prevalent (60.97%), followed by anxious attachment (24.39%), and disorganized attachment (14.63%). Regarding childhood trauma, the average total score on the Childhood Trauma Questionnaire (CTQ) was 56.34. Specifically, 39% of patients reported experiencing physical abuse, 24.4% reported sexual abuse, 14.6% reported emotional abuse, and 4.9% reported physical neglect. The Spearman correlation analysis between avoidant attachment and scores on the Childhood Trauma Questionnaire (CTQ) yielded a diverse set of findings. It indicated a significant positive correlation with physical abuse (ρ = 0.004, p < 0.001), a significant negative correlation with emotional abuse (ρ = -0.045, p < 0.001), a significant positive correlation with sexual abuse (ρ = 0.036, p < 0.001), a significant negative correlation with physical neglect (ρ = -0.083, p < 0.001), a significant negative correlation with emotional neglect (ρ = -0.047, p < 0.001), and a significant positive correlation with denial (ρ = 0.080, p < 0.001). On the other hand, the Spearman correlation analysis between anxious attachment and scores on the CTQ showed varying correlations: a significant positive correlation with physical abuse (ρ = 0.094, p < 0.001) and sexual abuse (ρ < 0.0001, p = 0.05).

**Conclusions:**

Our findings indicate that individuals with an insecure attachment style and a history of childhood trauma should be considered a high-risk group, necessitating early clinical intervention, continuous monitoring, and personalized therapeutic approaches designed to alleviate the psychological effects of trauma.

**Disclosure of Interest:**

None Declared

